# A Rare Intersection of Septic Cavernous Sinus Thrombosis and Subarachnoid Hemorrhage: Insights from the Case of a 70-Year-Old Patient

**DOI:** 10.3390/medicina60020253

**Published:** 2024-02-01

**Authors:** Chi-Ruei Li, Po-Han Chen, Se-Yi Chen, Tsung-Hsi Yang, Cheng-Siu Chang, Chao-Yu Shen, Fook-How Chan

**Affiliations:** 1 Department of Neurosurgery, Neurological Institute, Taichung Veterans General Hospital, Taichung 40201, Taiwan; fantastic1694@gmail.com; 2 Department of Neurosurgery, Chung Shan Medical University Hospital, No. 110, Section 1, Jianguo North Road, Taichung 40201, Taiwan; majicplayer@hotmail.com (P.-H.C.); sychen0102@gmail.com (S.-Y.C.); jimmyandpipi@hotmail.com (T.-H.Y.); chengsiu.chang@gmail.com (C.-S.C.); 3 Department of Medical Imaging, Chung Shan Medical University Hospital, Taichung 40201, Taiwan; shenchaoyu@gmail.com

**Keywords:** anticoagulant, cavernous sinus thrombosis, prognosis, septic emboli, subarachnoid hemorrhage (SAH)

## Abstract

We describe a rare and complex case of septic cavernous sinus thrombosis (SCST) in a 70-year-old patient who initially presented with ocular symptoms that rapidly progressed to severe intracranial vascular complications, including subarachnoid hemorrhage (SAH). Despite the use of broad-spectrum antibiotics and anticoagulants, the patient’s condition deteriorated. SCST, often caused by sinus infections, presents a significant diagnostic and therapeutic dilemma, with mortality rates exceeding 20%. This report underscores the diversity of clinical presentations, ranging from mild headaches to severe cranial nerve deficits, that complicate diagnosis and treatment. The inability to detect any aneurysms in our patient using magnetic resonance imaging (MRI) and computed tomography angiography (CTA) may indicate an alternative pathogenesis. This could involve venous hypertension and endothelial hyperpermeability. This case illustrates the need for personalized treatment approaches, as recommended by the European Federation of Neurological Societies, and the importance of a multidisciplinary perspective when managing such intricate neurological conditions. Our findings contribute to the understanding of SCST coexisting with SAH.

## 1. Introduction

Subarachnoid hemorrhage (SAH), a critical condition characterized by blood leakage into the subarachnoid space following the rupture of cerebral vessels, is associated with a high 30-day mortality rate of 40–50%. Despite significant advancements in diagnostics and treatment in recent decades, the management of SAH remains challenging. Aneurysmal rupture has been identified as the primary cause of mortality in over 80% of nontraumatic SAH cases [1]. The remaining cases, often linked to venous sinus thrombosis, present a rare yet grave diagnostic and therapeutic dilemma.

Patients with SAH who also have sinus thrombosis present with a wide array of symptoms ranging from relatively mild headaches to severe deficits in cranial nerve function. This diversity in clinical presentation significantly complicates both the diagnostic process and formulation of effective treatment plans. Several factors contribute to the development of SAH such as cerebral amyloid angiopathy, which may predispose individuals to vascular complications, and the presence of dural arteriovenous fistulae, which disrupt normal cerebral blood flow patterns. Septic emboli are notable concerns, especially in the context of systemic infections, given their potential to seed thrombotic material within the cerebral vasculature.

This case report meticulously delineates the diagnostic journey and management strategies employed for treating a 70-year-old patient who presented with a rare and complex clinical scenario of septic cavernous sinus thrombosis (SCST) concurrent with SAH. In addition to detailing the patient’s clinical progression and therapeutic interventions, this report provides a comprehensive exploration of the underlying pathogenesis, identifies potential risk factors, and discusses the broader implications for treatment in such intricate cases. We conducted an extensive review of the current literature (Table 1) to offer a holistic view that not only contributes to a deeper understanding of this specific case but also enhances the existing knowledge base surrounding the management of SCST complicated with SAH. In this report, we aimed to provide valuable insights for clinicians encountering similar challenging cases to help improve patient outcomes in this critical area of neurology.

## 2. Case Presentation

**Figure 1:**
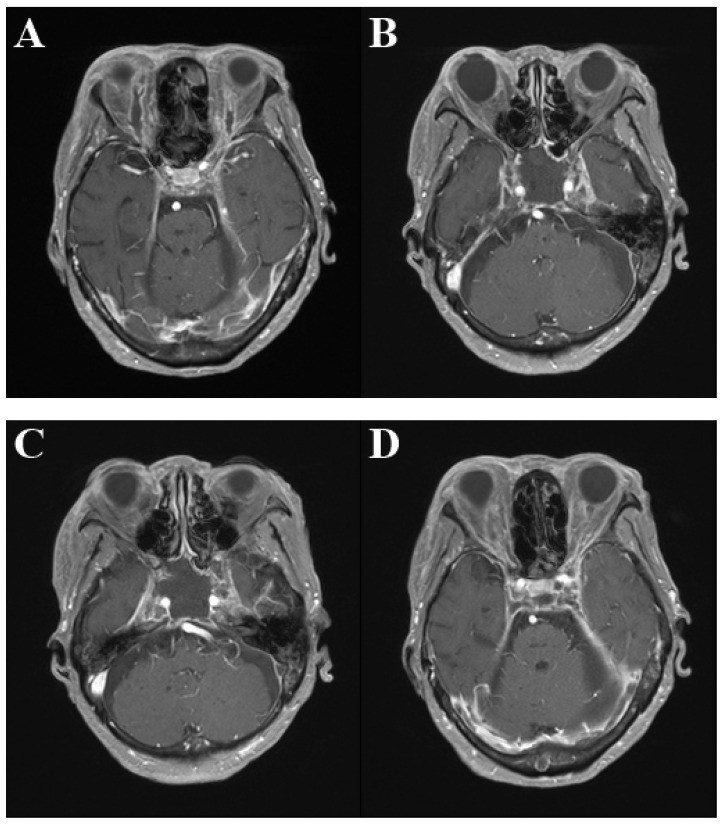
Brain magnetic resonance imaging with contrast enhancement. (**A**) Bilateral intraluminal sinus thrombosis and enlarged superior orbital vein; (**B**) filling defects in the bilateral cavernous sinus; (**C**,**D**) T1-weighted image with contrast revealed low signal intensity within transverse sinus and sigmoid sinus.

**Figure 2:**
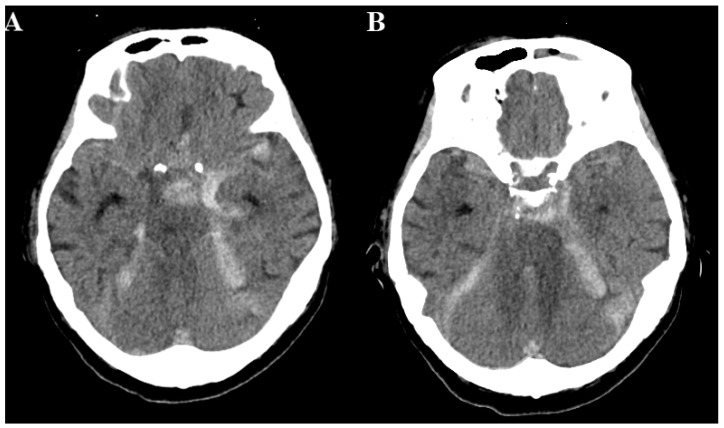
(**A**,**B**) Cranial computed tomography with contrast enhancement. Diffuse subarachnoid hemorrhage was noted.

A 70-year-old female patient was admitted to the hospital with painful ophthalmoplegia of the right eye and headache for 2 weeks. Associated symptoms, including nuchal pain and fever, were present prior to her visit. The patient had chronic diseases such as hypertension and a history of infarction of the left thalamus. Neurological examination showed limited ocular movement in the right eye but no pupillary abnormality. Blood analysis revealed markedly elevated levels of infection markers. Cerebrospinal fluid analysis through a lumbar puncture revealed bacterial infection of the central nervous system. Brain magnetic resonance imaging (MRI) revealed infiltrative contrast enhancement over the sphenoid sinus, bilateral cavernous sinus, and bilateral orbital region, compatible with infections or inflammatory processes. Engorgement of the bilateral ophthalmic vines compatible with cavernous sinus thrombosis was observed. A filling defect was also noted in the left transverse sigmoid sinus, and sinus thrombosis was suspected (Figure 1). No aneurysms or vascular malformations were found on cranial reconstructed magnetic resonance angiography. Two days later, the patient experienced a sudden change in consciousness on the Glasgow Coma Scale of E1V1M1, accompanied by the disappearance of the gag and corneal reflexes. The pupils showed inequivalent sizes with no detectable light reflex. Repeat cranial computed tomography angiography (CTA) showed diffuse SAH without a definite vascular lesion (Figure 2). Considering the patient’s current condition and poor prognosis, the patient’s family refused aggressive resuscitation. Eventually, the patient was declared clinically dead 3 days after the coma episode.

## 3. Discussion

SCST is an uncommon yet serious thrombotic condition that primarily originates from sinus infections. This pathology can also result from facial trauma, dental infections, complications following facial or transnasal surgery, or otitis media. In the context of an active infection, the primary clinical manifestations are ocular symptoms due to the involvement of the cranial nerves within the cavernous sinus. However, the more perilous aspects of this condition include complications such as meningitis, encephalitis, and brain abscesses, which represent the truly fatal elements of this disease. Additionally, progression to phlebothrombosis may precipitate severe hemorrhagic or infarct events, further complicating the clinical scenario.

In the present case, the patient initially presented with severe chemosis and painful ophthalmoplegia indicative of intraocular involvement. MRI confirmed the presence of an active infection in the cavernous sinus, with extension to the transverse and sigmoid sinuses. Within 48 h, the patient experienced a dramatic decline in consciousness accompanied by diffuse SAH. A review of the literature revealed that the concurrent manifestation of SCST and diffuse SAH is rare and has been documented in a handful of case reports. Similarly, Dolapsakis et al. [2] described an example of a sphenoid-sinusitis-related SCST that progressed to SAH and intraventricular hemorrhage. Notably, both cases showed a rapid and catastrophic decline, with fatal outcomes.

In contrast to clinical scenarios in which the rupture of mycotic aneurysms resulted in lethal hemorrhage, the current case showed an inability to detect any aneurysms in our patient using MRI, and computed tomography angiography (CTA) may indicate an alternative pathogenesis. However, the possibility of mycotic aneurysms cannot be entirely ruled out, as further invasive diagnostic procedures like digital subtraction angiography were not conducted, due to the patient’s clinical condition.

Upon reviewing the literature, we identified a potential pathogenesis involving the rupture of thin-walled, bridging subarachnoid cortical veins due to venous hypertension [7]. Additionally, in this case, the presence of an infectious and inflammatory environment further weakened the already fragile venous structures. This pathological condition, aggravated by oxidative stress, could lead to increased endothelial hyperpermeability, potentially culminating in a fatal hemorrhagic event [8].

The efficacy of anticoagulation therapy in both septic and aseptic cavernous sinus thrombosis (CST) remains a subject of debate, largely because of the lack of randomized controlled trials and small sample sizes in retrospective studies. An earlier meta-analysis highlighted the safety and improved outcomes associated with the use of subcutaneous low-molecular-weight heparin and intravenous unfractionated heparin. Weerasignhe et al. [5] recently reviewed cases of CST from 1980 to 2015; they found a reduction in mortality and enhanced recovery in the 41 patients treated with anticoagulation compared to in the 47 untreated patients. However, the risk of intracranial and systemic hemorrhages necessitates careful consideration of the timing and duration of anticoagulation therapy. The European Federation of Neurological Societies recommends a personalized approach to anticoagulation in cerebral venous and sinus thrombosis, suggesting a 3-month course for cases with transient risk factors and a 6–12-month course for idiopathic cases or mild thrombophilia [9]. Furthermore, during the treatment period, the targeted international normalized ratio should be maintained between 2.0 and 3.0 [10].

Despite advancements in diagnostic methodologies and the availability of broad-spectrum antibiotics, SCST remains a formidable intracranial infectious condition with a high mortality rate. Historical data from Southwick [11] and Yarington [12] indicate mortality rates of 50% and 80%, respectively. The recent literature on SCST, even in the antibiotic era, reports a mortality rate exceeding 20% [13]. In our patient, despite administering broad-spectrum antibiotics (ceftriaxone combined with vancomycin) for source control of the intracranial infection and implementing anticoagulant therapy, the patient’s condition continued to deteriorate significantly. This outcome underscores the complexity and severity of SCST and highlights the challenges in managing this life-threatening condition.

## 4. Conclusions

In this report, we present a rare case of SCST that initially manifested as intraocular symptoms, subsequently complicated by severe intracranial vascular complications. This case presented significant diagnostic and therapeutic challenges. We believe that this case merits a multidisciplinary discussion, focusing on both treatment modalities and pathogenesis, to enhance the understanding and management of such complex clinical scenarios.

## Figures and Tables

**Table 1:** Literature review of septic cavernous sinus thrombosis.

Author	Age (yrs)	Gender	Infection Source	Co-Morbidity	Organism	Antibiotic Choice	Anti-Coagulant	Surgical Treatment	Outcome
Sofferman et al. [2]	18	Male	Sphenoid sinusitis	N/A	Bacteriodes, Streptococcus intermedius, mixed anaerobe	Penicillin, Chloramphenicol, Aminoglycoside	Not used	Trans-septal sphenoidotomy	Full recovery
Weerasinghe et al. [3]	41	Male	Nasal furuncle	N/A	MSSA	Penicillin, Ceftriaxone, carbapenem, linezolid	Enoxaparin for 30 days	No	Full recovery
Sethi et al. [4]	25	Male	Nasal furuncle	Hepatitis C	MRSA	Vancomycin, Piperacillin/Tazobactam, Amphotericin-B	Unfractionated heparin	No	Expired
Dolapsakis et al. [5]	70	Female	Sphenoid sinusitis	Coronary artery disease, Diabetes mellitus	No finding	Vancomycin, Meropenem, Metronidazole	Enoxaparin for 2 days	No	Expired
Kotagiri et al. [6]	31	Female	Bacteremia	Methamphetamine abuse	MRSA	Vancomycin, Ceftriaxone, linezolid	Unfractionated heparin	No	Expired

Yrs: years; N/A: not applicable; MSSA: Methicillin-sensitive Staphylococcus aureus; MRSA: Methicillin-resistant Staphylococcus aureus.

## Data Availability

Data are contained within the article.
